# Formalin

**Published:** 1897-05

**Authors:** 


### Formalin.

For some time a need has been felt among physicians for some substance having the antiseptic powers of carbolic acid and bichloride of mercury, but which must neither be caustic nor corrosive. If the same substance could, in a measure, replace the clumsy heat-and-steam-sterilizing processes, so much the better. All this the water solution of formic aldehyde, known as formalin, will do, if present reports are true. When an alcohol is gently, oxidized an aldehyde is produced. This in turn may be oxidized to an acid. Formaldehyde ($C H_2O$) is the oxidation product of methyl alcohol, or wood spirit, and gets its name from the fact that its oxidation product is formic acid. Formaldehyde may be produced experimentally by passing the vapor of methyl alcohol, mixed with air, over a hot platinum spiral, when the odor will become noticeable. It is a very volatile liquid, which is quite soluble in water, and the usual commercial solution is about 40 percent. One of the characteristics of aldehyde is its property of uniting directly with other substances. It unites with hydrogen to form alcohol, with oxygen to form an acid, with ammonia to form aldehyde ammonia, and with other substances in the same way. Indeed, if it can find nothing else it will unite with itself to form more complex substances.

The disinfecting power of formalin is very great, and this gives it its great value. A 1-100000 solution will prevent the development of bacteria, while a 1-75000 is germicidal. A 5 percent solution gives better results than a $2\frac{1}{2}$ percent solution of carbolic acid or a 2-1000 bichloride solution.[1] The effect of a concentrated solution on living skin is like that of tannin, and prolonged application will cause necrosis. It has no detrimental action on metal or rubber. Uses for this substance will at once suggest themselves. It may be used for cleansing the hands, the seat of an operation, or infected wounds and cavities. It is an ideal substance for sterilizing instruments and keeping them sterile. In fact it may be used in every case in place of the bichloride.

Dr. W. S. Alexander,[2] of Oxford, Ohio, has given formalin considerable attention, and has used it in infectious diseases with good results. In diphtheria the air of the room was kept saturated with the vapor of formalin from a spray, and it was applied locally once or twice by means of cotton swabs. The false membrane entirely exfoliated in twelve hours. Whooping-cough was treated by spraying with a 1-percent solution. A $\frac{1}{2}$-percent solution, used as a spray, and inhalation of a 2-percent solution worked well in catarrhal troubles and in hay fever. By using a 40-percent solution chancroid and chancre healed rapidly after *one* application with no bad results except the temporary pain. The doctor has tried it in many other troubles with equally good results.

Outside of the fields of medicine proper a need of a good disinfectant has long been felt. Especially has this been true of our large public libraries. Books can not be treated with heat

1 Dr. De Buck and Dr. Vanderlinden-Ghent.

2 *N. Y. Medical Journal.*

or with steam, so it has been practically impossible to do anything with infected books but destroy them. Happily formalin comes to the rescue here also. It vaporizes easily, and has great penetrating powers. Elmer G. Horton, of the University of Pennsylvania, has experimented with formalin as a disinfectant for books, and has found that one cubic centimeter of formalin in three hundred cubic centimeters of air will perfectly sterilze a book in fifteen minutes. A longer time will not allow the admission of more air. In his experiments he used the bacilli of typhoid and of diphtheria, as being likely to occur in books. Some books containing papers smeared with cultures were placed *closed* in a bell-jar of known capacity, and a measured amount of formalin was allowed to evaporate spontaneously under the jar. It was allowed to stand some time, and then the papers and books were examined with the results stated above. This would be of easy application in a library. All that would be needed is a box of known capacity and a bottle of formalin. There is no action on the books.

Formalin has been in use for several years as a hardening and preserving agent for tissues, and its action in this line is much more satisfactory than that of alcohol. I have had an eye in a 2-percent formalin solution since June, 1896, which was enucleated by my father on account of a tumor within the ball. The eye is now firm and hard; there is not a wrinkle or any sign of shrinkage, and it is so clear that when held in front of an electric lamp one can look through the pupil and see the tumor *in situ.*

With the facts before him one can hardly help feeling that at last an ideal antiseptic and disinfectant has been found, and that the use of formalin in place of heat, steam, carbolic acid, and bichloride will in future have great use in all places where complete disinfection is desired.—*Carroll O. Southard, Stanford University, in Pacific Medical Journal, February,* 1897.

